# Diagnostic use of abdominal ultrasound in detecting extrapulmonary tuberculosis or lymphoma in an HIV-endemic region

**DOI:** 10.4102/sajhivmed.v26i1.1679

**Published:** 2025-03-21

**Authors:** Ellouise C. Adams, Katherine Antel, Jenna L. Bailey, Karryn L. Brown, Dharshnee R. Chetty, David Richardson, Estelle Verburgh

**Affiliations:** 1 Department of Clinical Haematology, Medicine, Faculty of Health Sciences, University of Cape Town, Cape Town, South Africa; 2 Department of Hematology Oncology, Faculty of Medicine, Medical University of South Carolina, Charleston, United States of America; 3 Department of Haematology, Faculty of Medicine, University of Cape Town, Cape Town, South Africa; 4 Department of Anatomical Pathology, Faculty of Health Sciences, University of Cape Town, Cape Town, South Africa

**Keywords:** HIV, lymphoma, tuberculosis, ultrasound, sub-Saharan Africa

## Abstract

**Background:**

Extrapulmonary tuberculosis (EPTB) is common among people living with HIV (PLWH). Abdominal ultrasound is an accessible investigation, frequently employed to support the diagnosis of EPTB, but may lead to misdiagnoses of diseases with overlapping clinical features, such as lymphoma.

**Objectives:**

To describe the abdominal ultrasound features and confirmed diagnoses of patients referred to a biopsy clinic with unexplained lymphadenopathy.

**Method:**

This was a retrospective descriptive study of patients attending the peripheral lymph node biopsy clinic at Groote Schuur Hospital between 2017 and 2020, who had abdominal ultrasound examinations while being investigated for unexplained lymphadenopathy. Ultrasound features were compared to the final diagnosis made on the lymph node biopsy.

**Results:**

Thirty-four patients were included, most of whom were PLWH (59%). Approximately one-third had a confirmed diagnosis of lymphoma (29%) and approximately one-third had a confirmed diagnosis of tuberculosis (32%). Splenic hypoechoic lesions were more common in patients with lymphoma (64%) than in patients with tuberculosis (46%) and malignancy (17%). Ascites was equally distributed between patients with tuberculosis (36%) and lymphoma (36%). The ultrasound report and confirmed diagnoses agreed in 40% of patients with tuberculosis. Additionally, 36% of patients with confirmed lymphoma were suspected to have tuberculosis based on the abdominal ultrasound.

**Conclusion:**

Abdominal ultrasound abnormalities such as splenic hypoechoic lesions, lymphadenopathy, and ascites/pleural effusion have a differential diagnosis including both tuberculosis and lymphoma, and should be investigated accordingly.

**What this study adds:** This study highlights the need to consider lymphoma in patients with ultrasound features such as splenic hypoechoic lesions, lymphadenopathy, and ascites, which are commonly considered diagnostic of EPTB.

## Introduction

The estimated HIV prevalence among the South African population is 12.7%. The total number of people living with HIV (PLWH) was estimated at 8.0 million in 2024.^1^ Extrapulmonary tuberculosis (EPTB) is more common in PLWH and is difficult to diagnose because of the paucibacillary nature of the disease.^2,3^ The diagnosis of EPTB in resource-limited settings is even more challenging. Without a proven diagnosis, physicians frequently turn to empiric tuberculosis therapy in tuberculosis-endemic areas.^4^ The careful follow-up of patients on empiric tuberculosis therapy and monitoring of their clinical response is important, but is seldom done in an overburdened healthcare system.^5^ Because of the high burden of patients with tuberculosis, as well as diagnostic limitations, physicians frequently use a composite of clinical findings and insensitive investigation findings combined into a ‘score’ to diagnose EPTB.^6,7^ Without close follow-up, this practice results in underdiagnosis of conditions that mimic tuberculosis.^8^ Missed, or delayed, diagnosis can be fatal for diseases such as cancer, where timely diagnosis affects outcomes. Cancer is the leading cause of death for PLWH in economically developed countries, and non-Hodgkin’s lymphoma is the most common cancer in the United States.^9^ The latest Global Cancer Observatory ranked lymphoma as the seventh most common cancer in South Africa.^7^ However, there are several challenges in studying cancer prevalence in PLWH in South Africa, and the true prevalence of lymphoma is likely higher than reported.^10^ A study done more than a decade ago in a tertiary centre in Johannesburg, South Africa showed that lymphoma has a 10–100-fold increased incidence in PLWH.^11^ Furthermore, the incidence of lymphoma is likely to increase as, even with antiretroviral therapy (ART), lymphoma has a 7.7–11.5-fold increased risk in PLWH, unlike other HIV-associate malignancies that are greatly reduced through early virological suppression.^12^

Lymphoma has many overlapping features with EPTB, including lymphadenopathy, fever, night sweats, weight loss, and abnormal abdominal ultrasound features.^13,14^ Cohort studies of patients with lymphoma from tuberculosis-endemic areas report up to 85% of patients on presumptive tuberculosis treatment at the time of lymphoma diagnosis.^3,15,16^

Point-of-care ultrasound (POCUS) is an accessible investigation, guiding healthcare workers in clinical decision-making. Its utility in detecting EPTB in PLWH has been well established.^17,18,19,20^ Features suggesting EPTB on abdominal ultrasound are hepatomegaly, hepatic lesions, lymphadenopathy, ascites, pleural effusions, splenomegaly, and splenic hypoechoic lesions.^18^ In PLWH, splenic hypoechoic lesions on ultrasound are present in one out of five patients with EPTB and their presence has, therefore, been suggested as a sufficient indication to initiate tuberculosis treatment in PLWH.^21^ However, presentation with extra-nodal lymphoma involving organs such as the liver and spleen is commonly seen in PLWH.^22^ Splenic involvement of lymphoma occurs in 30% – 40% of non-Hodgkin’s and one-third of Hodgkin’s lymphomas.^23^ Intra-abdominal lymphoma may frequently cause abdominal lymphadenopathy.^22^ Ascites is less frequent in lymphoma but may occur, especially in PLWH who have a higher rate of extra-nodal disease.^22^ Lymphoma is a commonly overlooked cause in the differential diagnosis of splenic hypoechoic lesions on ultrasound, due to the estimated 10 times higher incidence of tuberculosis compared to lymphoma in South Africa.^7,24^

The aim of this study is to describe the abdominal ultrasound features among patients referred to a lymph node biopsy clinic with unexplained lymphadenopathy, and to compare these features with their final diagnosis determined by a battery of tests including histology.

## Research methods and design

### Study design

This was a retrospective descriptive study. Study patients were identified from a cohort of patients referred to the rapid access diagnostic lymphadenopathy clinic (RADLAC) for lymph node biopsies from 01 November 2017 to 30 September 2020.

### Setting

The study was located at the Clinical Haematology unit, Groote Schuur Hospital (GSH), a tertiary referral academic hospital in Cape Town, South Africa.

### Study population and sampling strategy

Participants in the study were chosen from the RADLAC database if they had undergone an abdominal ultrasound at their referring healthcare facility, as part of the diagnostic work-up for unexplained lymphadenopathy, in the preceding 6 months. The inclusion criteria for the RADLAC were adults (≥ 18 years), referred with lymphadenopathy (lymph node > 20 mm in the widest diameter) located in either the cervical, axillary, or inguinal region. Patients on presumptive tuberculosis therapy were enrolled provided this had been given for less than 1 month. Patients with contraindications to core-needle biopsy (low platelets, other coagulopathy, clinically unstable, site of biopsy unsafe) were excluded.

### Data collection

As part of the routine enrolment of patients attending the RADLAC, demographic information, symptoms, symptom duration, physical findings, HIV status, blood results, and results of prior tuberculosis investigations performed, were recorded. The site of biopsy was recorded, along with other sites of lymphadenopathy. The presence and duration of constitutional symptoms (cough, loss of weight, night sweats, fever) were specifically enquired about, as was the duration of lymphadenopathy. Blood was taken for a full blood count with differential and lactate dehydrogenase (LDH) if not performed in the 2 weeks prior to lymph node biopsy. For all HIV-positive patients, a recent CD4 count and a viral load were collected for those on ART.

The folder numbers of all participants in the RADLAC study were utilised to verify whether an abdominal ultrasound had been performed as part of their diagnostic evaluation. Abdominal ultrasound reports were obtained from the Picture Archiving and Communication System (PACS), which contains imaging data, reports from GSH, and its referral hospitals. The ultrasounds were performed by sonographers, registrars, and qualified radiologists employed at different hospitals. The findings on the abdominal ultrasound report were recorded and added to the existing Research Electronic Data Capture (REDCap) database that is used to capture data for the RADLAC. The parameters that were reviewed on the abdominal ultrasound reports were hepatomegaly, hepatic lesions, lymph node size and location, presence of ascites or pleural effusion, splenomegaly, splenic hypoechoic lesions, and the suspected diagnosis made by the ultra-sonographer.

### Tests on lymph node specimens

In the RADLAC, fine-needle aspiration (using a 22-G needle and 5 mL syringe) was performed first to check for caseous material; if > 0.5 mL of caseous material was aspirated, the MTB/Rif Xpert Ultra assay (Cepheid, Sunnyvale, California, United States), an air-dried smear for acid-fast bacilli (AFB) using the Ziehl-Neelsen stain and a tuberculosis culture was performed.^3^ If < 0.5 mL of caseous material was aspirated, a core-biopsy was performed using an automated biopsy gun (BARD Magnum^TM^, CR Bard Inc., Covington, Georgia, United States) with a 14-G needle. The tissue underwent histological examination, which included AFB staining, the MTB/Rif Xpert Ultra assay, and tuberculosis culture. Additionally, it was submitted to the National Health Laboratory Services (NHLS) Anatomical Pathology Lab for morphological assessment and immunohistochemical staining. If all test results were inconclusive, the patient underwent either a repeat core-biopsy or an excision biopsy at the discretion of the treating clinician.^14^ As already described in the RADLAC, focused investigations were carried out on lymph node tissue and aspirate to diagnose tuberculosis and lymphoma.^14^ Tuberculosis was diagnosed on lymph node aspirate or tissue if AFBs were identified or if the culture or the Xpert MTB/RIF Ultra was positive. Bacterial adenitis was diagnosed if pus was aspirated, and all tuberculosis investigations were negative. Other diagnoses were made histologically on the core or excision biopsy. Lymphoma type was classified according to the 2016 World Health Organization (WHO) classification of lymphoma tissues.^25^

### Data analysis

Data were analysed using STATA V14 (Stata Corporation, College Station, Texas, United States).^26^ Categorical variables were described by frequencies and percentages and compared using Pearson Chi-squared or Fisher’s exact tests, as appropriate. Numerical variables were described by medians and interquartile ranges and compared using student’s *t*-tests (parametric data) and Mann-Whitney or Kruskal Wallis tests (non-parametric data). In exploratory analysis presented in Online Appendix 1, Table 1-A1, we calculated the diagnostic accuracy with 95% confidence intervals (sensitivity, specificity, positive predictive values, and negative predictive values) of selected individual and combined ultrasound features by comparing them to the composite reference standard of a histological, Xpert MTB/RIF Ultra or culture diagnosis. For all analyses, statistical significance was set at α = 0.05.

### Ethical considerations

Ethical approval for this study (reference no: HREC830/2020) and the RADLAC (reference no.: HREC674/2017) were obtained from the University of Cape Town, Faculty of Health Sciences’ Human Research Ethics Committee (HREC).Informed consent was obtained from all participants in the RADLAC study. This study was conducted in accordance with the Declaration of Helsinki.

## Results

**FIGURE 1:**
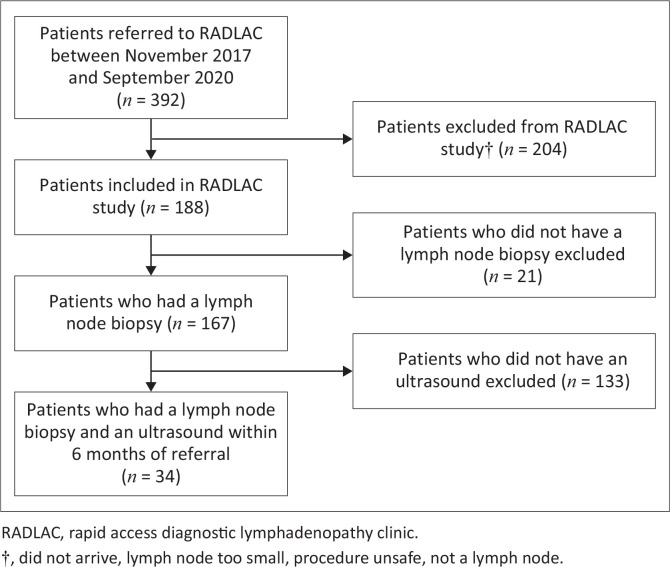
Consort diagram of patients included in this study.

Of the 188 patients included in the RADLAC study between November 2017 and September 2020, 34 (18%) patients had an abdominal ultrasound examination in their referring healthcare unit while being investigated for unexplained lymphadenopathy (Figure 1). Compared to those who did not have an abdominal ultrasound, the 34 patients who underwent abdominal ultrasound examination were significantly more likely to undergo chest X-ray (CXR) examination in their referring healthcare unit, (91% vs 38%, *P* < 0.001). Patients who had an abdominal ultrasound also had significantly lower haemoglobin (median 10.3 g/dL vs 12.4 g/dL, *P* < 0.001) and a higher proportion of axillary and inguinal lymphadenopathy (axillary: 18% vs 7%; inguinal: 9% vs 2%, *P* = 0.014), but a lower proportion of cervical lymphadenopathy (74%) compared to their counterparts (91%). There were no further significant differences between those who had an abdominal ultrasound and those who did not. Additional information on the 154 patients without an abdominal ultrasound is presented in Online Appendix 1, Table 2-A1.

The cohort with abdominal ultrasound had a median age of 33 years and a high prevalence of HIV positivity (59%) (Table 1). Among PLWH, 13 (65%) were on ART at the time of lymph node biopsy, 5 of whom were virally suppressed. The median CD4 count was 114 cells/mm^3^ (interquartile range [IQR]: 68–292). The most common biopsy site was the neck (74%). The median haemoglobin was significantly lower among PLWH compared to their counterparts (*P* = 0.013). There was no significant difference between median haemoglobin by diagnostic outcome (*P* = 0.473). The most common indication for an abdominal ultrasound was suspected EPTB (17/34 cases: 50%). Suspected lymphoma was only reported as the indication for ultrasound in three cases. Approximately one-third (29%) of patients had a final diagnosis of lymphoma and approximately one-third (32%) of patients had a diagnosis of tuberculosis.

**TABLE 1:** Demographic and clinical characteristics of patients with a lymph node biopsy and abdominal ultrasound between 2017 and 2020 (*N* = 34).

Variable	Total				HIV positive				HIV negative			
	*n*	%	Median	IQR	*n*	%	Median	IQR	*n*	%	Median	IQR
**Age at presentation (years)**	-	-	33.2	28.5–43.2	-	-	32.5	28.9–37.8	-	-	37.0	28.4–61.8
**Men**	13	38.2	-	-	8	40.0	-	-	5	35.7	-	-
**Blood results**											
Haemoglobin (g/dL)	34	100.0	10.3	6.5–12.2	-	-	8.2	6.3–11.2	-	-	11.8	10.8–13.6
Lymphocytes (× 10^9^/L)	28	82.4	1.5	0.8–2.2	-	-	0.9	0.7–1.7	-	-	1.8	1.1–2.4
Platelets (× 10^9^/L)	33	97.1	337	265–422	-	-	286	106–510	-	-	347	290–403
LDH (U/L)	29	85.3	270	191–340	-	-	262	201–340	-	-	272	183–343
**Constitutional symptoms present**	25	73.5	-	-	14	70.0	-	-	11	78.6	-	-
**CXR performed before biopsy**	31	91.2	-	-	20	100.0	-	-	11	78.6	-	-
**Lymph node site**											
Neck	25	73.5	-	-	15	75.0	-	-	10	71.4	-	-
Axillary	6	17.6	-	-	3	15.0	-	-	3	21.4	-	-
Inguinal	3	8.8	-	-	2	10.0	-	-	1	7.1	-	-
**Final diagnosis**											
Tuberculosis	10	29.4	-	-	5	25.0	-	-	5	35.7	-	-
Lymphoma	11	32.4	-	-	8	40.0	-	-	3	21.4	-	-
Metastatic malignancy	6	17.7	-	-	5	25.0	-	-	1	7.1	-	-
Other	5	14.7	-	-	1	5.0	-	-	4	28.6	-	-
Inconclusive†	2	5.9	-	-	1	5.0	-	-	1	7.1	-	-

Note: Total (*N* = 34; 100%), HIV positive (*n* = 20; 59%) and HIV negative (*n* = 14; 41%).

LDH, lactate dehydrogenase; CXR, chest X-ray; IQR, interquartile range.

† , final diagnosis described as inconclusive if no ultrasound diagnosis was indicated on the ultrasound report.

Table 2 describes the diagnostic relationship between the confirmatory testing (histology, microscopy, culture, cytology, Xpert MTB/RIF Ultra) and the suspected diagnosis on abdominal ultrasound. The finding on the ultrasound report was ‘inconclusive’ in most cases (*n* = 20; 59%) and lymphoma was never suspected as the diagnosis. The ultrasound report was deemed inconclusive when no diagnosis was suggested on the report by the reporting clinician. The ultrasound report and the confirmed diagnosis were consistent in only 40% of the patients diagnosed with tuberculosis. Additionally, 36% of patients with confirmed lymphoma were suspected to have tuberculosis based on the abdominal ultrasound. At the time of attending the RADLAC, 13 patients (38%) were on empiric tuberculosis therapy. Of these, only five patients had a final diagnosis of tuberculosis, while the others were diagnosed with lymphoma (*n* = 5), metastatic malignancy (*n* = 1) and reactive lymphadenopathy (*n* = 2).

**TABLE 2:** Diagnostic relationship between abdominal ultrasound findings and confirmed diagnosis from lymph node biopsy.

Suspected diagnosis based on abdominal ultrasound	Confirmed diagnosis from lymph node biopsy									
	Tuberculosis (*n* = 10)		Lymphoma (*n* = 11)		Metastatic malignancy (*n* = 6)		Other† (*n* = 5)		Inconclusive (*n* = 2)	
	*n*	%	*n*	%	*n*	%	*n*	%	*n*	%
Tuberculosis (*n* = 11)	4	40.0	4	36.4	1	16.7	2	40.0	0	0.0
Lymphoma (*n* = 0)	0	0.0	0	0.0	0	0.0	0	0.0	0	0.0
Metastatic malignancy (*n* = 1)	0	0.0	0	0.0	1	16.7	0	0.0	0	0.0
Other (*n* = 2)‡	0	0.0	1	9.1	0	0.0	0	0.0	1	50.0
Inconclusive§ (*n* = 20)¶	6	60.0	6	55.6	4	66.7	3	60.0	1	50.0

Note: Diagnostic tests on lymph node specimens included histology, microscopy, culture, cytology, and Xpert MTB/RIF Ultra tests.

† , Other biopsy diagnoses included: 3 reactive, 1 sarcoidosis, 1 sinus histiocytosis;

‡ , The two other diagnoses made on ultrasound were gallstones;

§ , Final diagnosis described as inconclusive if no ultrasound diagnosis was indicated on the ultrasound report;

¶ , Of the 20 inconclusive abdominal ultrasounds, 18 had abnormalities present, while 3 were normal.

Regarding specific ultrasound findings presented in Table 3, splenic hypoechoic lesions (46%) and lymphadenopathy (43%) were most reported. Splenomegaly was not present in patients with tuberculosis but was seen in 36% of lymphoma patients. Hepatic lesions (4%) were the least common ultrasound finding. Splenic hypoechoic lesions were more common in patients with lymphoma (64%) compared to patients with tuberculosis (46%) and malignancy (17%). Lymphadenopathy was most often found in lymphoma patients (64%), followed by patients with malignancy (50%) and tuberculosis (18%). Ascites was equally distributed between patients with tuberculosis (36%) and lymphoma (36%).

**TABLE 3:** Abdominal ultrasound findings present among patients diagnosed with tuberculosis, lymphoma, or malignancy on lymph node biopsy.

Abdominal ultrasound finding	Lymph node biopsy diagnosis					
	Tuberculosis (*n* = 11)		Lymphoma (*n* = 11)		Malignancy (*n* = 6)	
	*n*	%	*n*	%	*n*	%
Hepatomegaly (*n* = 6; 21.4%)	2	18.2	3	27.3	1	16.7
Hepatic lesions (*n* = 1; 3.6%)	0	0.0	0	0.0	1	16.7
Lymphadenopathy (*n* = 12; 42.9%)	2	18.2	7	63.6	3	50.0
Ascites (*n* = 9; 32.1%)	4	36.4	4	36.4	1	16.7
Pleural effusion (*n* = 6; 21.4%)	3	27.3	1	9.1	2	33.3
Splenic hypoechoic lesions (*n* = 13; 46.4%)	5	45.5	7	63.6	1	16.7
Splenomegaly (*n* = 6; 21.4%)	0	0.0	4	36.4	2	33.3
3 features present (lymphadenopathy, ascites/pleural effusion and splenic hypoechoic lesions) (*n* = 5; 17.9%)	2	18.2	3	27.3	0	0

Note: Diagnostic tests on lymph node specimens included histology, microscopy, culture, cytology, and Xpert MTB/RIF Ultra tests.

## Discussion

This study aimed to provide a descriptive analysis of abdominal ultrasound findings in patients who were comprehensively investigated for lymphadenopathy and to compare these features to their confirmed lymph node biopsy diagnosis. Our study highlights the risk of underdiagnosis of lymphoma due to overlapping radiological features between tuberculosis and lymphoma, specifically splenic hypoechoic lesions. Considered a key diagnostic feature of tuberculosis, these lesions could not reliably distinguish between tuberculosis and lymphoma, whether used alone or in combination with other ultrasound features such as ascites, pleural effusions, and lymphadenopathy. We found splenic hypoechoic lesions in 46% of tuberculosis patients and in 64% of lymphoma patients. Interestingly, splenomegaly emerged as a potential discriminator between the two conditions, being absent in all tuberculosis patients but present in 36% of those with lymphoma.

A systematic review evaluating the ultrasound features of PLWH with tuberculosis coinfection in five countries, including developed and developing countries, showed splenic hypoechoic lesions were present in 63% of patients. First, in comparison, our study identified splenic hypoechoic lesions in 46% of patients with tuberculosis and in 64% of patients with lymphoma.^21^ Although smaller, our study included HIV-infected and HIV-uninfected patients with tuberculosis or histologically proven lymphoma in South Africa, providing a critical nuance to the application of their results that ensures lymphoma diagnosis is not overlooked. Second, our study showed the combination of three ultrasound features – ascites/pleural/effusions, lymphadenopathy, and splenic hypoechoic lesions – was also non-specific, observed in 18.2% of tuberculosis patients and 27.3% of lymphoma patients. It is known that one of the weaknesses of POCUS in the diagnosis of tuberculosis is poor specificity.^27^ Consequently, diagnostic mimics like lymphoma are missed in a tuberculosis-endemic area.^28^ Griesel et al. conducted a prospective study of HIV inpatients, with WHO danger signs and cough, in a South African hospital.^17^ They examined multiple ultrasound features among PLWH with and without tuberculosis co-infection and found that using a combination of three ultrasound features (ascites/pleural effusions, lymphadenopathy and splenic hypoechoic lesions) was highly specific (98.9%) for diagnosing tuberculosis. All three features were present in 11% of their cohort, but they did not include patients with lymphoma as a sub-group for analysis. Our data indicates that although a combination of markers may improve specificity, it does not reliably exclude lymphoma.

Discrepancies between our findings and the above studies may also be due to the use of different reference standards, different abdominal ultrasound criteria, and different populations. Our study included HIV-positive and HIV-negative individuals, and we used multiple diagnostic tests including histology, microscopy, culture, cytology, and Xpert MTB/RIF Ultra. Studies included in the systematic review did not all make use of newer tuberculosis diagnostic tests such as the Xpert MTB/RIF Ultra and largely did not carry out proper histological confirmation of available tissue for exact diagnosis, including lymphoma. This may have ultimately resulted in misdiagnosis of patients. Nonetheless, we suggest that abnormal findings noted on an abdominal ultrasound should prompt more focused investigational techniques to confirm a diagnosis and facilitate appropriate treatment and patient management. Additional investigations might include flow cytometry on pleural or ascitic fluid to assess for a clonal lymphoid population, or an ultrasound guided intra-abdominal lymph node biopsy if there is not an accessible peripheral lymph node.^29^

The novelty of this small pilot study is the use of both histology and Xpert MTB/RIF Ultra as part of the battery of diagnostic tests. Our study highlights the risk of underdiagnosis of lymphoma due to overlapping clinical and radiological features between tuberculosis and lymphoma. In a tuberculosis-endemic area, it is important to confirm the diagnosis of tuberculosis. In the context of patients started on empirical tuberculosis therapy, patients should be followed up and monitored for the improvement of symptoms and weight gain. Patients with persistent lymphadenopathy and constitutional symptoms on empirical tuberculosis therapy should be referred for urgent biopsy and histological diagnosis. In settings where a lymph node biopsy is not possible, an Xpert MTB/RIF Ultra on needle aspirates of lymph nodes has been shown to perform well and can confirm tuberculosis without a biopsy.

Our study has limitations. First, it was conducted in a highly selected, small population of patients referred for unexplained lymphadenopathy, presumably after other investigations were inconclusive. This resulted in a study that was underpowered to evaluate diagnostic accuracy and may have introduced bias by enriching for lymphoma. Second, retrospective procurement of ultrasound reports from four different referring hospitals meant that ultrasound reporting could not be standardised. Third, all participants had an accessible peripheral lymph node; therefore, our findings may not be generalisable to patients with tuberculosis or lymphoma without the presence of peripheral nodes. Despite these limitations, the use of a stringent reference standard (histology, microscopy, culture, cytology, Xpert MTB/RIF Ultra) as the comparator to ultrasound findings has generated robust data with important implications. Additionally, the study design was strengthened by all biopsies being performed in a single centre and by a single, experienced clinician.

These findings suggest that the abdominal ultrasound should not be used to differentiate between lymphoma and tuberculosis, as they have overlapping radiological features. The ultrasound is nevertheless a valuable tool and should be used to identify patients who are at high risk of having either tuberculosis or lymphoma, and to prompt further investigation to confirm the diagnosis. Patients not responding to empiric tuberculosis therapy should be referred early for lymph node biopsy. Further research is required to validate these findings in a primary care setting and refine a pragmatic diagnostic pathway that minimises inaccurate and late diagnosis. The apparent value of splenomegaly as a rule-out test for tuberculosis also requires further investigation as this may be a valuable approach to identify patients in whom urgent investigation is required to exclude malignancy.

## Conclusion

Abdominal ultrasound is widely used to support the diagnosis of EPTB in tuberculosis-endemic areas. Our findings highlight that this practice may lead to significant underdiagnosis of lymphoma as many ultrasound features considered pathognomonic of tuberculosis are frequently seen in lymphoma. This emphasises the necessity of obtaining a definitive diagnosis in patients with lymphadenopathy to ensure prompt and appropriate therapy to improve patient outcomes.
