# Corrigendum: Diagnostic use of abdominal ultrasound in detecting extrapulmonary tuberculosis or lymphoma in an HIV-endemic region

**DOI:** 10.4102/sajhivmed.v26i1.1736

**Published:** 2025-06-09

**Authors:** Ellouise C. Adams, Katherine Antel, Jenna L. Bailey, Karryn L. Brown, Dharshnee R. Chetty, David Richardson, Estelle Verburgh

**Affiliations:** 1 Department of Clinical Haematology, Medicine, Faculty of Health Sciences, University of Cape Town, Cape Town, South Africa; 2 Department of Hematology Oncology, Faculty of Medicine, Medical University of South Carolina, Charleston, United States of America; 3 Department of Haematology, Faculty of Medicine, University of Cape Town, Cape Town, South Africa; 4 Department of Anatomical Pathology, Faculty of Health Sciences, University of Cape Town, Cape Town, South Africa

In the original published article, Adams EC, Antel K, Bailey JL, et al. Diagnostic use of abdominal ultrasound in detecting extrapulmonary tuberculosis or lymphoma in an HIV-endemic region. S Afr J HIV Med. 2025;26(1):a1679. https://doi.org/10.4102/sajhivmed.v26i1.1679, the authors wish to also acknowledge the funder, the Cancer Association of South Africa (CANSA).

Instead of:

Funding information

Research reported in this publication was supported by the Fogarty International Center and National Heart, Lung and Blood Institute of the National Institutes of Health under Award number D43 TW010345.

It should be:

Funding information

Research reported in this publication was supported by the Fogarty International Center and National Heart, Lung and Blood Institute of the National Institutes of Health under Award number D43 TW010345 and the Cancer Association of South Africa (CANSA).

The authors apologise for this error. The correction does not change the study’s findings of significance or overall interpretation of the study’s results or the scientific conclusions of the article in any way.

